# A qualitative study on negative experiences of social media use and harm reduction strategies among youths in a multi-ethnic Asian society

**DOI:** 10.1371/journal.pone.0277928

**Published:** 2022-11-22

**Authors:** Ellaisha Samari, Sherilyn Chang, Esmond Seow, Yi Chian Chua, Mythily Subramaniam, Rob M. van Dam, Nan Luo, Swapna Verma, Janhavi Ajit Vaingankar

**Affiliations:** 1 Research Division, Institute of Mental Health, Singapore, Singapore; 2 Department of Psychosis, Institute of Mental Health, Singapore, Singapore; 3 Saw Swee Hock School of Public Health, National University of Singapore, Singapore, Singapore; 4 Departments of Exercise and Nutrition Sciences and Epidemiology, Milken Institute School of Public Health, The George Washington University, Washington, DC, United States of America; University of New South Wales, AUSTRALIA

## Abstract

**Purpose:**

This study aimed to expand and inform the emerging body of research on the negative experiences of social media use among youths and how youths deal with them, in an Asian setting, using a qualitative approach.

**Methods:**

Data were collected using 11 focus group discussions (FGDs) and 25 semi-structured interviews (SIs) among youths aged 15 to 24 years residing in Singapore who were recruited via purposive sampling. Data were analysed using thematic analysis.

**Results:**

The salient negative effects mentioned by participants include the development of negative reactions and feelings from upward comparisons with others (e.g., others’ achievements and lifestyle), receiving hurtful comments, exposure to controversial content (e.g., political events and social movements), as well as the perpetuation of negative feelings, behaviours, and sentiments (e.g., rumination, unhealthy eating behaviour, and self-harm). Participants also described strategies which they have employed or deemed to be useful in mitigating the negative effects of social media use. These include filtering content and users, taking breaks from social media, cognitive reframing, and self-affirmation, where they identify and change stress-inducing patterns of thinking by setting realistic social, physical, and lifestyle expectations for themselves, and focusing on self-development.

**Conclusion:**

The current results highlight that while youths experience negative effects of social media use, they have high media literacy and have employed strategies that appear to mitigate the negative effects of social media use. The findings can inform various stakeholders involved in helping youths navigate the harms of social media use or provide directions for intervention studies aimed at reducing the harms of social media use.

## Introduction

The marked rise in social media use today exemplifies the evolution of the digital landscape. Social media platforms such as Facebook and YouTube continue to dominate the online scene with an estimated 2.9 and 2.1 billion monthly active users respectively [1, 2]. Other platforms such as Instagram and TikTok, developed later, have since gained traction, especially among younger audiences with 120 and 100 million active users respectively [3–5]. Using social media sites can arguably be a norm of growing up in the digital age, especially among youths [6]. This is reflected in research conducted in 2019 among the younger population of the US where 85% of teenagers were reported using YouTube, while 72% used Instagram and 69% used Snapchat [7].

Social media platforms have drastically changed the way people socialize, share information, present themselves, perceive others, and work [8]. It is an influential and integral element in today’s interaction and communication which is readily available and easily accessible through multiple devices such as smartphones, computers, and tablets. Furthermore, persistent cues through notifications and variable reward mechanism (e.g., social validation for social media post through likes and comments, infinite scroll etc.) encourage greater social media use. Naturally, this phenomenon has sparked interest among researchers to examine the experiences of social media use, particularly among youths given new social dynamics [9] and the intricate period of transition to adulthood where young people are experiencing significant developmental and psychosocial shifts including identity exploration [10, 11]. It is also a period where youths are experiencing intensified peer relationships, seeking romantic relationships, and engaging with potential or current partners [10] and may be motivated to gain attention from their peers or to observe peers’ self-presentations.

Arguably, the harms or benefits of social media use depends on how and why people use them, and who uses them. Social media sites provide opportunities for youths to develop and maintain social relationships [12], cultivate a sense of belongingness, present themselves to others [13], keep up to date [14] and even learn about sexual health and identity [15]. Some studies have reported associations between social media use and psychological well-being, which is a state of wellness where an individual is feeling good and functioning well based on having positive relationships with others, environmental mastery, self-acceptance, personal growth, autonomy, sense of purpose in life and experiencing positive emotions such as happiness and contentment [16, 17]. Specifically, judicious use of social media has been found to be associated with several positive psychosocial outcomes such as increased quality of friendship [16] and social support [17]. On the other hand, social media use has also been associated with negative experiences such as stress, social isolation, cyberbullying, and mental health issues including depression, anxiety, poor body image and disordered eating [18], or found to have no substantive links to mental health issues [19–22]. For example, a longitudinal study by Heffer et al. [20] found that social media use did not predict future depressive symptoms and adolescent girls who experience depressive symptoms tend to use more social media across time, and not vice versa. In another longitudinal study by Coyne et al. [19], increased time spent on social media was not associated with increased depression or anxiety across adolescents’ developmental period at an individual level.

The literature on social media use has been primarily focused on the association between social media use and its impact on users’ well-being, while factors associated with harm reduction or how young people manage or reduce the harms of its use have received less attention. Notwithstanding, spending less time on social media sites or using social media mindfully was perceived by youths to help mitigate the negative experiences from social media use [23, 24]. In addition, high levels of confidence, high level of media literacy and appreciation of individual differences appeared to mitigate the potential negative effects of social media exposure on body image among adolescent girls [25]. Findings from these studies highlight some perceived effective means of mitigating the negative experiences of social media use. Nonetheless, given the dearth of research on harm reduction strategies for negative experiences of social media use, the current study aimed to contribute to the emerging evidence base on negative experiences of social media use among youths and ways youths reduce the harms of social media use in Asia. Identifying harm reduction strategies is critical as it offers insights into how young people mitigate these negative effects which can inform the development and implementation of interventions aimed at improving health outcomes. A qualitative approach will allow a deeper understanding of the psychological mechanisms and processes (i.e., how and under what circumstances) behind youths’ negative experiences and the ways they deal with them. Thus, we used a qualitative approach to explore the broad experiences of young people in Singapore with social media use to answer the following questions– 1) What are the negative experiences of social media use? 2) What are the strategies youths employ to reduce these harms?

## Methods

### Sample

A purposive sampling design was used to obtain the study sample of young people aged 15–24 years, with an approximately equivalent proportion of men and women as well as those belonging to age groups 15–19 and 20–24 years, and three main ethnic groups in Singapore (Chinese, Malay and Indian). Initially, referrals for these participants were sought from colleagues and acquaintances who were provided with the study brochures. Subsequently, participants who had participated were also given the study brochures to disseminate to others and initiate snowball recruitment. Efforts were also made to include young people who had experiences of psychological distress, school drop-out or risky behaviours (e.g., substance use, gang involvement, and incarceration) to ensure greater diversity of the study sample. Referrals for these participants were sought from community-based youth welfare organisations which are providing services to clients with these experiences. Written informed consent was obtained from all participants before they participated in the study. For participants aged below 21 years, parental consent was also obtained. Ethical approval was obtained from the National Healthcare Group’s Domain Specific Review Board (DSRB No. 2020/0228).

### Data collection

Data were collected through a combination of semi-structured interviews (SIs) and focused group discussions (FGDs) conducted in English and via online videoconferencing using the Zoom platform (11 FGDs, 21 SIs) or in person (4 SIs) between May 2020 and November 2020. Almost all SIs and FGDs were conducted by the lead female researcher [JAV] who has extensive experience in conducting qualitative studies, while some were conducted by the other trained study team members [ES1, SC, ES2, and YCC] who have also received training in qualitative research and had prior experience conducting qualitative interviews. Data from this study originates from a larger study which examined youths’ interpretation of positive mental health and its associated pathways. As part of that study, participants were asked questions relating to social media use (as seen in Table 1) and their mental health including their or their peers’ experience of any pleasant or unpleasant experiences/incidents on social media, their feelings arising from those experiences/incidents and how it influenced their or their peers’ mental health. Interviews ceased once data saturation was reached, where no new information was observed and collected. The present study explored data gathered on the harms of social media use and the strategies youths employ or perceive to be useful against these harms.

**Table 1:** Interview guide to examine the harms of social media and strategies youth adopt to mitigate those harms.

• Tell me about your experience of using social media.
• What are some of the social media platforms that you often use?
• What do you or your friends usually do on these platforms? How does it make you/your friends feel?
• What about your friends? Any pleasant or unpleasant experiences/ incidents that you can recall in relation to social media? How does it make them feel?
• What are some of the strategies you use when engaging with social media?
• You mentioned that social media can affect [experience]. How can you or one overcome this? What are some of the strategies you can use to maintain positive mental health? Activity (only for FGDs): “When I use social media, I feel…”
• When did you/your friends feel [experience/emotion]? Can you/someone describe any such incident or experience? How did it influence your/their mental health?

### Data analysis

All FGDs and SIs were audio-recorded and transcribed verbatim. Data management and coding were conducted on NVivo 11 software. Data were analysed using thematic analysis as informed by Braun and Clark [26]. Following the inductive approach, five study team members [ES1, SC, ES2, YCC, JAV] identified preliminary codes on the harms of social media use and strategies to avoid those harms from the first six transcripts using the open coding method [27]. Through thorough and iterative discussions within the study team, codes were generated into higher-order concepts (themes and sub-themes) based on their common properties and formed an initial codebook. Regular discussions were carried out to review, refine and build consensus on the final codebook, which served as a framework for coding of remaining transcripts [28]. A high level of consensus was reached during the framework development process.

## Results

Data from the present study comprise 36 data units– 11 FGDs and 25 SIs. A total of 95 young people (51 women and 44 men; mean age = 20.1 years) participated in the study. Participants belonged to Chinese (n = 32), Malay (n = 27), Indian (n = 28) and other ethnicities (n = 8) such as Filipino or Burmese. Among them, eight participants had a history of psychological distress, school drop-out or risky behaviour (e.g., substance use, gang involvement and incarceration). Table 2 displays information on participants’ sociodemographic backgrounds.

**Table 2:** Sociodemographic characteristics of the study population.

		Focus group discussions (n = 11; 70 participants)		Semi-structured interviews (n = 25)		Total	
**Age, years (mean±SD)**		19.8**±**2.5		21.0**±**2.6		20.1**±**2.5	
		**n**	**%**	**n**	**%**	**n**	**%**
**Gender**	**Women**	37	52.9	14	56.0	51	53.7
	**Men**	33	47.1	11	44.0	44	46.3
**Ethnicity**	**Chinese**	25	35.7	7	28.0	32	33.7
	**Indian**	20	28.6	7	28.0	27	28.4
	**Malay**	21	30.0	7	28.0	28	29.5
	**Others**	4	5.7	4	16.0	8	8.4
**Education**	**Primary**	1	1.4	3	12.0	4	4.2
	**Secondary**	33	47.1	3	12.0	36	37.9
	**Junior College**	12	17.1	10	40.0	22	23.2
	**Diploma**	12	17.1	4	16.0	16	16.8
	**ITE** ^**1**^	6	8.6	2	8.0	8	8.4
	**Tertiary**	6	8.6	3	12.0	9	9.5
**Employment**	**Employed, full-time**	4	5.7	3	12.0	7	7.4
	**Employed, part-time**	6	8.6	6	24.0	12	12.6
	**Unemployed, never worked**	18	25.7	4	16.0	22	23.2
	**Unemployed, past work experience/internship**	42	60.0	12	48.0	54	56.8

^1^Institute of Technical Education

### Social media use

All participants reported having experience with social media use. The primary purposes of using social media included sharing information, gathering information, connecting with others, maintaining relationships, and entertainment which occurred on various platforms including Instagram, Facebook, TikTok, Reddit, Twitter, and YouTube. The following results are presented as themes encapsulating the various negative experiences and impact of social media use as experienced and perceived by participants or their peers, as well as practised and perceived mitigations against these harms.

### Negative experiences of social media use

*Theme 1*: *Experience of negative emotions and behaviour from upward comparisons with others*. A common theme in the interview was the development of negative reactions from upward comparisons with others. Comparisons with others’ achievements (e.g., school-related, work-related), material possessions, and experiences (e.g., travels), were more saliently discussed during the interviews as opposed to physical appearance, which was noticeably mentioned more by female participants than male participants. Participants noted that comparisons were usually made with peers, social media influencers, and celebrities with narratives on comparisons to peers and social media influencers being more pronounced. The resulting negative reactions to these comparisons included the development of negative feelings (e.g., feelings of inferiority, insecurity, self-consciousness, hurt, and loneliness), negative behaviours (e.g., self-loathing, starving, engaging in unhealthy competitions and overly intense workouts), and negative body image (e.g., idealizing skinny body types).

“*Social media is a platform where there are many many different beauty standards*, *and I can’t help but tend to compare myself with them*. *I think over the past one year or so*, *I tried many*, *many*, *many ways to lose weight because I felt that I wasn’t good enough to the point that like I lost too much weight which wasn’t good for myself*.*”–FGD03*

“*When you’re looking at like*, *maybe the Instastory of like*, *some influencer or like*, *and stuff like that*, *or people who are like*, *on holidays and whatnot*, *then kind of make you feel as though like*, *you are inadequate or you are missing out or you’re not doing something right*, *that kind of puts on unnecessary trigger or stress on you*.*”–SI10*

Furthermore, comparisons with others can result in the erosion of individuality when users feel compelled to follow the trend or be like other users just to fit in. This desire to belong can inadvertently fuel feelings of insecurity.

“*… it’s very natural to then feel*, *"Okay*. *We must do this as well because that’s what a lot of our generation people are trying to do*.*" We’re trying to follow other people*. *We’re trying to follow to be like someone else*. *And along that process*, *we either worsen the insecurities we already have or we don’t feel confident about ourselves*. *We don’t feel good about ourselves*. *We don’t want to be ourselves”–FGD10*

*Theme 2*: *Experience of negative emotions from receiving hurtful remarks*. Some participants recounted being emotionally affected by hurtful remarks directed towards them or having witnessed their friends suffer from such insults. These hurtful remarks stem from varying sources, including disagreement with or disapproval towards the content (e.g., activities, opinions, or comments) they had shared, and come from both known and unknown contacts. Furthermore, the anonymity afforded by fake social media accounts allows unbounded and harsh criticisms without consequences towards the perpetrator. Following that experience, some removed the content shared or deactivated their accounts.

“*One of my friends*, *she posted her point of view about this situation and other things*. *Then there’s this another person who just created another fake account and went on to talk shit about her and attack her saying that "Oh*, *you shouldn’t have this point of view*. *Why are you acting this way*,*" all that stuff*. *Then it sort of affected her mental health… she was really affected by it that she had to deactivate her account for a couple of weeks until she got back online again–SI14*

“*I removed them [family] but they somehow managed to stalk me still*. *They started talking about me–that I’m sharing my personal life in social media and disgracing the family*. *So*, *they caught me and I had to remove all of the videos because it was really very stressful for me*. *That’s one of the bad experiences*.*”–SI21*

A participant also recounted how easy it was to have been a bully on social media:

“*…talking to people online through the screen is so much easier*. *So*, *it also increases cyberbullying*. *Yeah*, *it’s so easy to cyberbully someone because it’s just like… and then just send*. *Yeah*, *I’m sorry to say this*, *but I was a bully also*. *Yeah*, *it felt very bad*, *but I’m not that person anymore*.*”–SI07*

*Theme 3*: *Experience of negative emotions from exposure to controversial content*. Some participants described experiencing or seeing their peers experience negative feelings from exposure to controversial content. While a broad range of content was discussed, the prominent ones leaned towards global political events (e.g., repression of Uyghurs), natural or man-made disasters (e.g., Beirut explosion), social issues (e.g., misogynistic content and animal cruelty), and social movements (e.g., Black Lives Matter). Consumption of this controversial content had left them feeling disturbed, drained, helpless, overwhelmed, and pessimistic.

“*I see a lot of my friends online*, *they might overdo it and it might like start to drain them (be)cause they tend to watch a lot of such disturbing videos and- so I think last week*, *I was speaking to someone and they were saying they feel very upset*, *and I asked them why*. *They were like they were watching a lot of animal cruelty videos*, *and it really affect them a lot”–SI08*

“*…I feel like certain situations are full of injustice*, *for example*, *the Uyghur situation or how people criticize Muslims a lot in this world*. *And it just frustrates me because a lot of*, *yeah*, *misunderstandings are due to them not wanting to educate themselves*. *Like the comments are so ignorant and it’s just so frustrating*.*”–FGD07*

*Theme 4*: *Perpetuation of negative emotions*, *behaviours*, *and sentiments*. The perpetuation of negativity through social media use was commonly mentioned by participants. First, social media platforms enable rumination through posts or stories. Second, negative sentiments are sometimes echoed by like-minded communities and exacerbated. Third, social media can encourage the perpetuation of negative behaviours such as self-harm, unhealthy eating behaviour and unhealthy coping styles when users follow self-harm content/accounts and emulate them.

“*If they’re feeling down right*, *instead of looking at something positive*, *they end up going on Reddit*, *and they look at—they enter those communities that are all the same people*, *and it becomes an echo chamber of negative things*, *and it gets worse because it’s just the same kind of people*.*” FGD08*

“*It’s very*, *very sad because you see that their (friends with eating disorders) whole lives are just engrossed and obsessed with numbers*, *calories*, *food*, *exercise*, *all these things*. *And it’s really very*, *very*, *very sad and disheartening to see that social media has such a huge role in worsening these things*.*”–SI16*

“*I work mostly with youths at risk*, *and the kind of content that you follow online is always more towards the emo genre*. *Sometimes*, *it does have the elements of suicide or taking a life*, *or like self-harm*, *and all those various aspects*, *and that became their way of dealing with stress*, *either they learn to take the action from there*, *or it just comes ingrained*, *and they do something similar towards the destructive and that’s maladaptive*.*”–FGD08*

### Mitigation of negative effects of social media use

*Theme 5*: *Filtering content and users*. Most participants described curating content or being selective of users they follow as means to reduce any negative effect social media use can have on their mental health. These include choosing only positive content to follow from the start, filtering out accounts followed (e.g., unfollowing accounts which trigger negative feelings), muting selected users’ posts/stories on social media, and scrolling past negative content.

“*I don’t really feel that way [negative feelings] when I use social media because*, *yeah*, *why would I want to make myself feel so bad*? *Yeah*, *I tend to go for the more positive kind of posts instead of those that kind of puts you down”–FGD01*

“*Let’s say you have a favourite YouTuber who is so pretty*. *She’s a beauty vlogger*. *And you love watching her videos*. *But every time you watch her videos*, *you feel so self-conscious about your own face*. *I think it’s important to make that hard sacrifice to cut it out*, *to stop watching that YouTuber*.*”- SI13*

*Theme 6*: *Taking breaks from social media*. Participants also highlighted the importance of being aware of the negative impacts of social media consumption on themselves, such as from social comparisons or absorbing overwhelming content and managing time spent on social media to reduce these impacts. They also mentioned different ways and benefits of taking breaks from social media use. For some, breaks can either be temporary or permanent (e.g., deactivating social media accounts for good), and the benefits include being able to focus on other tasks at hand (e.g., studying/schoolwork) and experiencing boosted mental health.

“*…I think what we don’t realize is that these (social media) are outlets that feed on your energy every single day*. *So*, *if it gets to a point where you can’t function or perform well because of it*, *then you need to give yourself a break from it*.*”–FGD7*

“*So*, *for one period*, *I deleted all my social media like kind of a social media cleanse*, *and I think a lot of youth do that as well*, *as they are growing a little bit older*, *because social media is very superficial*, *in a sense*.*”–FGD2*

*Theme 7*: *Cognitive reframing and self-affirmation*. This theme explicates the conscious effort by users to identify and change stress-inducing patterns of thinking by setting realistic social, physical, and lifestyle expectations for themselves and not being swayed by unrealistic social media portrayals. Participants also showed awareness of the superficial nature of the content on social media which does not necessarily mirror complete real-life situations or experiences. In addition, focusing on self-development and self-affirmation were other ways participants noted as safeguards against the pitfalls of social comparisons on social media.

“*… we need to have a stronger sense of reality and understand that social media is really not everything*. *Like what [participant] said*, *there’s a backstage and there’s a front stage that you want to portray*, *and no one wants to show their dirty laundry on social media*. *They want everything to be good*.*”–FGD4*

“*So*, *you see these people’s successes… but then I just try to remind myself that*, *"Okay*. *I’ll be happy for them*. *Yours will come in time*,*" instead of feeling like*, *"Oh*, *why isn’t mine here*?*”–SI03*

“*I don’t regularly tell positive affirmations to myself*, *but maybe once in a while*, *I will just look in the mirror and say positive things about myself*, *and it actually makes me feel better immediately*, *and I think that has an impact on my mood*”–*FGD08*

## Discussion

Findings from this study expand and inform the emerging body of research on the negative experiences of social media use among a sample of youths in Asian society and how they deal with them. While youths reported experiencing or witnessing their peers experience significant negative effects of social media use, it is promising to note youths’ awareness of these negative effects and their attempts to avoid or reduce them.

Youths’ narratives suggest a predisposition towards upward social comparisons, particularly with peers and influencers on social media sites. This tends to result in negative effects; most of which are reflected in prior studies including the development of negative feelings (e.g., feelings of inadequacy, lowered self-esteem [29]), as well as an unhealthy mindset (e.g., negative body image [30]) and behaviours (e.g., disordered eating behaviours [31]). [32, 33]. Scholars suggest that the fundamental and universal desire for comparisons with others serves a variety of functions such as evaluating the self [34], fulfilling affiliation needs [35], and being inspired [36]. Given the social functions of social media sites and the detailed information about others, it may be natural for people to engage in social comparisons either consciously or unconsciously [37]. Furthermore, unlike real-life situations, social media sites allow people to present an optimized or idealized version of themselves and their experiences [38, 39]. It is therefore possible that further exposure to ‘enhanced’ profiles can create more discrepancy between their perceived self and others and perhaps amplify feelings of inadequacy. This could be supported by the finding of Chou and Edge [40] who examined the impact of using Facebook on people’s perception of others’ lives and found that people who have spent more time on Facebook tend to perceive other social media users as having better lives than they do.

Qualitative studies examining comparisons on social media among young people in Western populations have mostly focused on examining the relationship between social media use and physical or bodily appearance [25, 41, 42] or found the appearance-related social comparison to be discussed by participants when examining the role of social media on mental health [43]. In such studies, negative impacts from such comparisons were often highlighted. For example, in a study based in the UK by Easton et al. [42], the authors examined young adults’ (aged 18–25) experience with ‘fitspiration’ (blend of “fitness” and “inspiration”) on social media. They found that comparisons with another’s perceived fitness (content on healthy lifestyle habits, relating to exercise and diet), can give rise to negative effects on their psychological health. In particular, minor negative effects include being frustrated about the deceptive nature of posts and jealousy towards unattainable body appearance while more perturbing effects include negative feelings towards their bodies and unhealthy eating habits. These findings are reflected in the narratives of a few participants in this study, particularly female participants, who had mentioned engaging in upward physical appearance comparisons and resonated with such experiences. On the other hand, a unique finding which emerged from this study is the upward comparison with others’ achievements (e.g., academics, employment) or material possessions and lifestyles. Research exploring cultural differences in social comparisons suggests that people living in countries whose cultures tend to be more collectivistic, rather than individualistic, are more likely to engage in social comparisons [44] and that Eastern cultures are suggested to be more concerned about one’s relative social standing [45]. Therefore, it seems unsurprising that youths in Asian society are inclined to seek upward social comparisons with peers’ achievements, such as having a good social network, occupation, and education, which are contributors to a person’s social standing.

Participants highlighted cognitive reframing as a strategy to reduce the harms of social comparisons on social media. Cognitive reframing among participants entails identifying negative patterns of self-evaluations and reinterpreting how they view content on social media, such as reminding themselves of the superficial nature of social media portrayals as well as creating more realistic self-expectations. Research indicates that low self-esteem has been linked to unrealistic standards for self-evaluation [46] and that negative self-evaluations can occur when discrepancy increases between ideal and real self-image [47]. In the context of social media, for example, exposure to ‘fitspiration’ content, which tends to involve images and messages praising thinness and high fitness levels [48, 49], can lead to increased body dissatisfaction if these ideals are internalized and unattained [39] Cognitive reframing mentioned by youths, such as setting realistic body image or achievement expectations, can therefore act as a buffer against the development of negative self-evaluations when comparing with unrealistic and unattainable body images or others’ achievements on these platforms.

Narratives of negative feelings from exposure to controversial content are worth paying attention to. Specifically, youths mentioned feeling drained and helpless from bearing witness to cruelty or disasters to which they are unable to contribute to improving the situation. Social media has emerged as a source of news content over the years [50] and provides many opportunities to be exposed to news incidentally or deliberately through content shared by others within their social networks [51], or when they follow official accounts of news broadcasters. Media writers have argued that news in the digital age has become increasingly visual, with images taken from various sources, and written to convey excitement and danger, and be fear-laden [52]. Importantly, user-generated images of important world events are frequently captured on smartphones by witnesses in these events, allowing the audience to view such events in real-time [53]. Such user-generated images allow news broadcasters to display more intense and shocking visuals that may not have been available or approved in earlier times. It is therefore unsurprising that there is an observed negative effect on mental well-being with exposure to such raw and intense visuals and commentaries.

However, it is arguably reassuring to note that participants are aware of the control they have over social media’s influence on their lives and have exercised proactive self-control strategies to regulate their social media use, such as limiting content seen on social media or keeping off social media. Participants recognize the feeling of relief and benefit to their mental well-being when they step away from stress-inducing content or when they disabled their social media accounts, incidentally, highlighting the pervasiveness of social media in their lives. Indeed, some studies have shown that taking a break from social media positively affects subjective well-being [54–56]. It is also worth noting that keeping off social media among participants appears to involve internal negotiation and contention, alluding to resisting compulsions towards using it.

The negative experiences and harm minimisation strategies reported in this study align well with some emerging initiatives to protect young people from the harms of social media use. An example of such an initiative is #Chatsafe. #Chatsafe was developed to guide and educate young people about communicating safely about suicide on social media [57, 58]. The social media campaign was found to be effective in improving young people’s capacity to intervene against suicide online, perceived internet self-efficacy and safety when communicating about suicide on social media [58]. A similar initiative based on the #Chatsafe guideline was also implemented in Singapore [59]. Specifically, the #Chatsafe guideline was adapted to the local context of Singapore into a #PauseBeforeYouPost campaign which educates young people on how to conduct safe conversations around mental health issues and engage safely with those who are at risk of suicide. In addition, a #Chatsafe training curriculum is also being developed for youths and caregivers to provide them with the relevant skills and knowledge to engage positively with suicide-related online content and support those around them who are in distress. The effectiveness of this campaign can inspire the implementation of future interventions targeting different needs of youths, as exemplified by the narratives among this sample population, to mitigate negative experiences and outcomes of social media use. For example, users could be taught and reminded of proper etiquette when communicating with others on social media to avoid making hurtful remarks. In addition, self-esteem, which can mediate the effects of upward comparisons on well-being, could also be addressed in these campaigns.

Efforts could also be extended beyond the social media realm as programs in schools. Educators could teach youths how to manage content and conversations on social media and encourage the diversification of content by suggesting accounts that nurture intellectual passions or interests, resilience, and increase self-esteem in them. As exemplified by narrations of adolescents from Burnette et al.’s [25] study, a supportive school environment and its effective communication of social media-related messages and programmes on accepting differences in body image can contribute to the development of high media literacy and confidence among them.

### Strengths and limitations

This study has several strengths. Data were gathered from both FGDs and SIs, and deviant samples, allowing for the generation of broad and rich qualitative data. FGDs are generally more dynamic and allow participants to discuss and expand on their pre-existing ideas in light of points mentioned by other participants, which may not have been uncovered in in-depth interviews [60]. On the other hand, SIs allows for the gathering of greater insight into the individuals through the discussion of topics in detail [60]. However, study findings must be considered in the context of several limitations. The nature of the topic–the negative impact of social media use–may be sensitive or controversial to some participants; therefore, participants may limit sharing due to social desirability bias [61]. In addition, we did not examine whether experiences of social media use differ across individuals with different patterns of social media use, or those from different sociodemographic and sociocultural backgrounds, which may account for differences in experiences of negative effects and subsequently adoption and success of different coping mechanisms. It is recommended that future research delve into this topic further and explore the interaction between an individual’s socioecological environment and their experiences with social media use. Future research could also investigate differences in terms of strategies adopted by Asian and Western youth populations.

## Conclusion

Results from the present study indicate that social media can influence youths’ lives today. While social media can enhance learning, connection and communication [62], the salience of its negative effects on users’ mental well-being drives the need to actively monitor these harms and explore effective ways to steer users away from them. The current results offer a preliminary portrait of the salient negative effects of social media use in a multi-ethnic Asian youth population. It also indicates that while youths experience the negative effect of social media use, they have high media literacy and have employed strategies that appear to mitigate the negative effects of social media.
